# Addressing Health Disparities through Community Engagement in Artificial Intelligence-Driven Prevention Science

**DOI:** 10.1007/s11121-025-01863-2

**Published:** 2026-01-14

**Authors:** Emily E. Haroz, Novalene Goklish, Adrienne Dillard, Roy Adams, Sheana S. Bull, Ricardo F. Gonzalez-Fisher, Pamela Valenza, Spero M. Manson, Roland J. Thorpe

**Affiliations:** 1 Center for Indigenous Health, Johns Hopkins Bloomberg School of Public Health, Baltimore, MD 21205 USA; 2 Centers for American Indian and Alaska Native Health, Colorado, School of Public Health , University of Colorado Anschutz Medical Campus, Aurora, CO 80045 USA; 3 Kula No Na Po`E Hawaii, 2150 Tantalus Drive, Honolulu, HI 96813 USA; 4 Department of Psychiatry and Behavioral Sciences, Johns Hopkins School of Medicine, Baltimore, MD 21287 USA; 5 Colorado School of Public Health, University of Colorado Anschutz Medical Campus, Aurora, CO 80045 USA; 6 Servicios de La Raza, Denver, CO 80216 USA; 7 Tepeyac Community Health Center, Denver, CO 80216 USA; 8 Program for Research On Men’s Health, Hopkins Center for Health Disparities Solutions, Johns Hopkins Bloomberg School of Public Health, Baltimore, MD 21205 USA

**Keywords:** Artificial intelligence, Community engagement, Machine learning

## Abstract

Artificial intelligence and machine learning (AI/ML) in prevention science may improve or perpetuate health inequities. Community engagement is one proposed strategy thought to empirically mitigate bias in AI/ML tools. We outline how to incorporate community engagement at every stage of the model development and implementation. Borrowing from a framework for phases of prevention research, we describe the value and application of engaging communities to help shape more rigorous and relevant applications of AI/ML for prevention science. We provide concrete examples from real-world applications, including efforts in suicide prevention with Indigenous communities, on chronic disease prevention for Hispanic and Latino populations, and a community-driven effort to leverage AI/ML to improve allocation of resources focused on social determinants of health for Native Hawaiians. This work aims to provide applied examples of how community-engagement has been incorporated into AI/ML development and implementation, with the goal of encouraging those in the prevention science field to consider the voices of the community as the use of such tools grows. Engaging with the community around AI/ML is critical to ensure these tools reach populations in need and advance health equity for all.

“No kakou, na kakou”" — "“for us, by us.”

## Introduction

The rapid growth of use of artificial intelligence (AI) and machine learning (ML) is transforming public health approaches on a global scale. These technologies are being extensively implemented to enhance efficiency and quality of health services. AI/ML approaches are now being used to enhance risk identification, augment intervention development and training, and for many other purposes (Amisha et al., 2019; Shaheen, 2021). AI/ML approaches offer unique advantages for prevention science including the ability to process large datasets to identify population-based insights, tailored interventions based on a variety of risk profiles or contexts, and to support delivery of prevention services at scale (Amisha et al., 2019).

Artificial intelligence (AI) encompasses any computational approach designed to mimic human cognitive functions, while machine learning (ML) represents a specific subset that learns patterns from data without explicit programming. Examples of other AI approaches include rule-based systems, generative models, and natural language processing. The necessity of AI/ML in prevention science stems from fundamental limitations of traditional approaches. Prevention science increasingly confronts complex, multi-factorial health challenges that often require commensurate methodological approaches. While, there has been increasing attention paid to potential study designs to address this complexity (Lich et al., 2013), less has been focused on the underlying data analytic methods. Traditional analytic methods struggle to identify non-linear relationships and interaction effects among social determinants, behavioral factors, and health outcomes that drive population-level disparities. AI/ML approaches address these limitations by enabling analysis of complex, high-dimensional data to identify at-risk populations earlier, personalize prevention strategies based on individual risk profiles, and predict intervention responsiveness across diverse contexts—capabilities essential for advancing precision prevention at scale (Fishbein & Dariotis, 2019).

However, these powerful capabilities introduce unique risks. Recent years have seen a surge in use of AI/ML models in public health and prevention science, accompanied by a growing realization of the need for quality assurance when using these approaches (Food (Food 2019)). However, as these tools are more widely applied, biases that lead to inequities in services have become apparent. In their paper examining a widely used AI/ML tool to guide the provision of care to the sickest patients, Obermeyer et al., 2019 found that, using an AI/ML-generated risk score, Black patients were considerably sicker than White patients, at the same level of risk score, but were less likely to receive needed care (Obermeyer et al., 2019). In another example Coley et al., 2021 showed that a suicide risk model used in a large healthcare system vastly underperformed in American Indian/Alaska Native (AI/AN) patients compared to other racial and ethnic groups (Coley et al., 2021).

While bias in healthcare and preventative services extend beyond AI/ML-based tools, these tools can inherit and amplify bias in unique ways (Chen et al., 2021; Ferryman & Pitcan, 2018). Barriers to study participation and healthcare access, and differences in care-seeking behaviors result in datasets that overrepresent certain groups (Gianfrancesco et al., 2018; Hing & Burt, 2009; Weber et al., 2017). Accumulated disparities and biases in measurement of outcomes result in biased prediction targets that reflect different levels of need for different patients (Mullainathan & Obermeyer, 2021). Metrics used to optimize and evaluate algorithms may obscure when models fail to work well for subpopulations (Subbaswamy et al., 2021). Even sound algorithms may result in bias if problems or deployment settings are not chosen equitably.

Researchers have suggested several strategies to help advance equity issues in AI/ML applications in health. Substantial work has gone into mathematical formalizations of fairness (Mehrabi et al., 2022), as well as quantitative and qualitative methods to audit algorithms for bias (Chen et al., 2018; Moons et al., 2019; Subbaswamy et al., 2021; Wang et al., 2022). Efforts are underway to diversify the pool of publicly available health data (Daneshjou et al., 2022; Mapes et al., 2020), as well as the pool of AI/ML researchers (AIM-AHEAD, n.d.); Shanker, 2022). Some authors have pointed to the importance of regulatory oversight to mitigate bias and ensure fairness (Rajkomar et al., 2018; Thomasian et al., 2021). Berdahl and colleagues (Berdahl et al., 2023) in their review identified strategies to advance health equity including improving data quantity and quality, evaluating disparities, increasing model transparency, enhancing governance, and involving the community.

A growing body of literature acknowledges the importance of community engagement to the equitable development of precision approaches to public health and medicine (Dolan et al., 2023) and improving machine learning model’s rigor and relevance (Birhane et al., 2022; Kulynych et al., n.d). Yet, there remains little concrete guidance on how to achieve effective community engagement in AI/ML development processes. This paper addresses this gap by providing a phase-based framework for systematically integrating community engagement throughout AI/ML development in prevention science. Drawing from established prevention science phases and contemporary community engagement principles (Leve et al., 2024); Boyd et al., 2023), we demonstrate how community voice can strengthen rigor, relevance, and equity at each stage—from conceptualization through sustainability. We illustrate this framework through real-world applications with American Indian, Hispanic, and Native Hawaiian communities, showing how different engagement approaches can be adapted to local contexts while maintaining core equity principles. Ultimately, this work aims to move beyond theoretical acknowledgment of community engagement’s importance to practical guidance for prevention scientists developing AI/ML tools.

## Community Engagement to Advance Health Equity

Advancing health equity is a priority across prevention initiatives, with community engagement serving as a cornerstone strategy. Community engagement exists on a continuum with increasing levels of involvement, impact, trust, and communication flow, progressing from outreach to consultation, involvement, collaboration, and finally to shared leadership (Leve et al., 2024). This approach requires moving beyond university-driven research agendas toward mutually defined or even community-driven agendas, emphasizing cultural humility in creating “mutually respectful, equal, and dynamic partnerships between academic and underrepresented communities” (Leve et al., 2024).

Community engagement and Community-Based Participatory Research (CBPR) represent distinct but complementary approaches. While community engagement encompasses a spectrum of involvement levels, CBPR is a specific research orientation that “equitably involves community members, organizational representatives, and academic researchers in all aspects of the research process” with shared decision-making throughout (Collins et al., 2018). Both approaches are effective in reducing health inequities (Ortiz et al., 2020) and are considered the “gold standard for prevention science that advances health equity” (Boyd et al., 2023).

Successful community engagement can significantly improve the relevance, rigor, and reach of research (Balazs & Morello-Frosch, 2013). Stewart et al. (2015) and Pratt and de Vries (2018) highlight the importance of community involvement in research design and implementation, particularly as it enhances acceptability and increases willingness to participate in research studies. Similarly, recent articles highlight the importance of community engagement in responding to the COVID-19 pandemic, including helping to reduce mortality from the disease (Haroz et al., 2022; Manson & Buchwald, 2021; Santibañez et al., 2019).

The integration of AI/ML tools in prevention science introduces unique equity challenges that require explicit community engagement strategies. Public health institutions must “prioritize equity in AI design and implementation, minimizing the risk of reinforcing existing health disparities” through training datasets that are “inclusive and representative of diverse populations” (Panteli et al., 2025). Fisher and Rosella (2022) identify explicit consideration of equity and bias as one of six strategic priorities for successful AI implementation, emphasizing the need to “carefully evaluate and assess potential sources of bias throughout development” and “engage with the community” as essential practices. Without community engagement, AI systems risk perpetuating mistrust from marginalized communities (Leve et al., 2024).

Effective community engagement in AI/ML development requires sustained investment in building trust and capacity. This includes ensuring community members have input into research processes and recognizing that “without a history of positive collaboration with communities…researchers miss individual representation, but also their input” (Leve et al., 2024). Contemporary approaches must incorporate explainable AI technologies to “ensure decisions made by AI systems are understandable and fair” (Panteli et al., 2025), while fostering transparent partnerships that address community priorities and values throughout the development lifecycle.

## How Can Community Engagement be Used to Develop AI-Based Prevention Science Tools?

While existing literature recognizes the importance of community engagement in AI/ML development (Berdahl et al., 2023; Birhane et al., 2022), there remains a critical gap in systematic frameworks for prevention science applications. Our work addresses this gap by providing a phase-based framework illustrated through real-world case studies, demonstrating how community engagement can be integrated throughout AI/ML development in prevention science contexts.

**Fig. 1:**
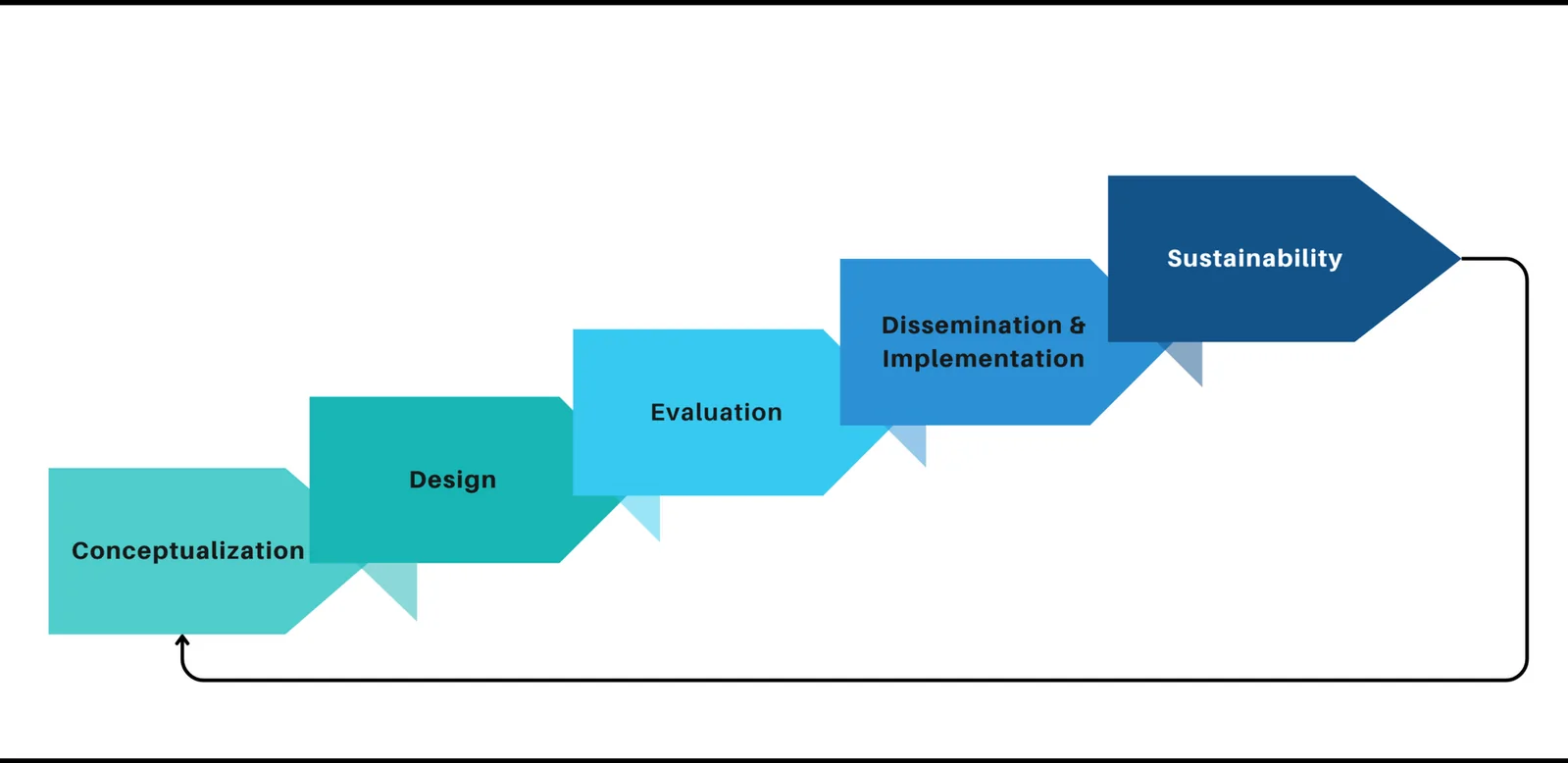
Phases of prevention research for AI/ML supported strategies

Drawing from the phases of prevention research (Fig. 1), we propose viewing AI/ML development through similar stages including: (1) conceptualization, (2) design of prevention strategy, (3) efficacy testing, (4) effectiveness testing, (5) dissemination and implementation, and (6) sustainability. Community engagement strengthens each stage, ensuring AI-assisted preventative interventions address genuine needs while remaining responsive to community needs.

During the Conception Phase, engagement with communities facilitates identifying priority health conditions that require attention and the contextual factors that may impact appropriate utilization of the tool. Short-term engagement strategies employed at this phase include establishing a community advisory board (CAB), understanding baseline AI literacy, or conducting stakeholder interviews. Long-term engagement strategies involve providing training in data science or related fields to empower community members with the necessary skills to develop and guide their projects effectively.

In the Design Phase, community input can shape the tool’s user interface, functionality, and planned workflow to make these domains more intuitive, accessible, and acceptable for diverse users or for a particular group of users. This phase may involve working with stakeholders to determine how to convey the tool’s information in an accessible and appropriate manner. Again, short-term strategies may involve user-centered design cycles or stakeholder interviews. Longer-term solutions may include developing and investing in infrastructure to make this process easier and more efficient.

Moving into the EvaluationPhase, community engagement helps ensure that appropriate data are included, results are interpreted consistent with community values and perspectives, and the tool is helpful for specific population segments through priority subgroup analyses. Evaluation results should be reviewed with advisory boards and local stakeholders. Further questions from community can be asked of the evaluation data to answer key questions prior to implementation. Here, short-term and long-term engagement strategies mirror earlier phases and include additional training that could help adapt these methods to community contexts.

During the Dissemination and Implementation Phase, insights from the community can guide strategies for effective dissemination and implementation to ensure that the community receives the benefits. Community-engaged implementation science tools and strategies may be particularly useful in this phase (Berdahl et al., 2023).

The Sustainability Phase encompasses two critical dimensions often overlooked in AI/ML literature. Implementation sustainability involves the organizational, financial, and governance structures needed to maintain tools over time. Algorithmic sustainability addresses a unique challenge: maintaining model performance as underlying disease distributions change due to intervention. This dual challenge is particularly complex in community-engaged contexts where algorithmic updates must preserve community trust and oversight. Continuous community feedback remains essential to assess long-term impact, audit for performance disparities and safety, make decisions about algorithmic updates, and ensure continued community benefit.

By integrating community engagement throughout these stages, the processes used to develop AI/ML-enabled tools become more inclusive, responsive, accurate, and responsible, ultimately leading to equitable and impactful innovations across all segments of the population. Ultimately it is a cyclical process that allows for further refinement of tools to ensure the highest quality approaches being delivered in the service of prevention.

## Example of Community Engagement to Develop and Launch an AI/ML Suicide-Risk Tool

Here we highlight the development and implementation of the Native-RISE (Risk Identification and Enhanced Care) system to illustrate how community engagement, framed within the phases of prevention, can be used throughout the AI/ML tool development and implementation process. Suicide rates among American Indian/Alaska Natives (AI/ANs) are disproportionately high, a trend that has worsened over time (CDC (Centers 2021); Stone et al., 2023). While many AI/ML suicide-risk models exist, almost none have been developed specifically for AI/ANs, a gap that the Native-RISE project aims to address (Cwik et al., 2016).

**Fig. 2:**
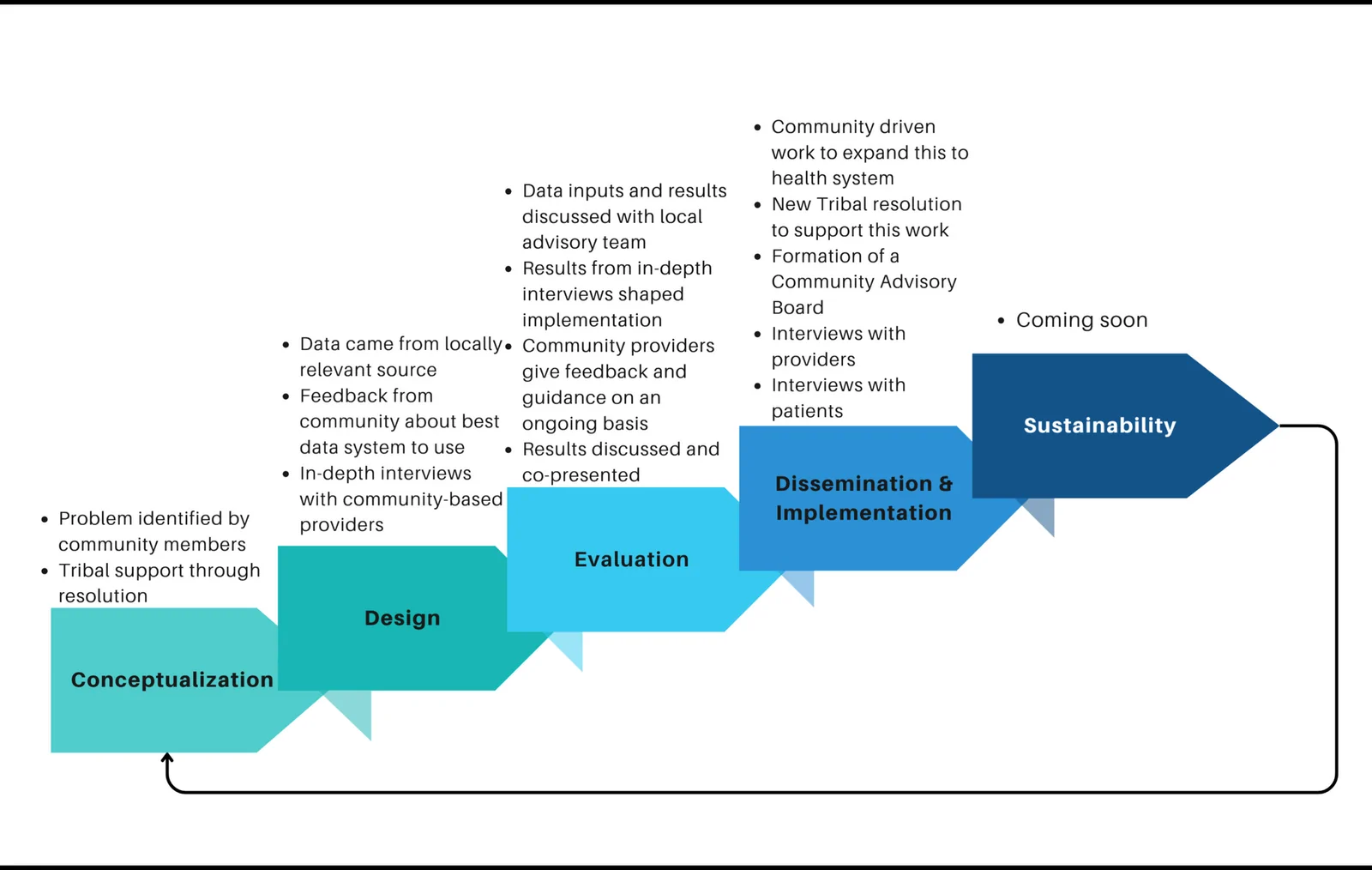
Community engagement in Native-RISE as applied to the phases of prevention research for AI/ML supported strategies

In 2017, tribal partners worked with long-standing university partners (Johns Hopkins Center for Indigenous Health; JHCIH) to support development of a tool to identify individuals at high risk in the Native community. Working with JHCIH researchers, members of the community obtained a tribal resolution to support analysis of local data to help in this process (Fig. 2, conceptualization). AI/ML tools were selected as a potential method to meet the community’s needs.

The resulting work examined 10 years of local surveillance data to identify those at risk of suicide attempt within 12 months of their last referral for suicide related behaviors to a local suicide surveillance and case management system. Results indicated that a machine learning algorithm (Logistic Regression based with 73 features) using these data produced an Area Under the Curve (AUC) of 0.87 (95% CI ± 0.04). This substantially out performed a comparison of basing risk high-status on a past suicide attempt, our best known single risk factor indicator and what was being used in practice (AUC = 0.57; 95% CI ± 0.08) (Haroz et al., 2019). While this was a significant improvement, translation to practice was key (Fig. 2, design phase). We interviewed program staff, asking how they currently evaluated risk and how this type of tool could be helpful. Generally, staff welcomed this tool, indicated preferences for it to show a high vs. low-risk classification, and discussed how much of their risk assessment is done through their clinical observations in real time, rather than past history, when meeting with a person. Information from how staff approach suicide risk informed workflow and how to set values to define high- vs. low-risk groups. In this instance, case managers favored specificity (meaning fewer false positives) so that that risk model was trustworthy and with the understanding that case managers’ clinical judgment would compensate for some loss in sensitivity (e.g., fewer false negatives) (Fig. 2; Efficacy Phase) (Haroz et al., 2021).

In the next step, (Fig. 2, Evaluation Phase), we evaluated the algorithm’s performance in real time. We conducted a prognostic study of the model performance at identifying those at risk of another suicide-related event among 400 individuals. The risk model continued to improve risk identification, was associated with an increase in reach of care for individuals at highest risk, and when the model was used by staff, helped to reduce the risk of suicidal behaviors for these highest-risk cases (Haroz et al., 2023). Results of all of these studies were disseminated with manuscript review and approval coming from the Tribe. Subsequent dissemination (e.g., conferences, papers) included people from the community who represent a critical voice in how this work was conducted and its implications locally and for the broader scientific field. Finally, based on these results and ongoing communication with other community partners, we sought and obtained an external grant to expand this work to the health system. During the upcoming Implementation phase (Fig. 2), we are working with the Indian Health Service, the provider of health services to the more than 2.2 million AI/AN people who are members of federally recognized tribes, to expand the reach of this work (R01MH128518). As the model is rolled out, ongoing monitoring will include bi-annual reviews of model performance disaggregated by key demographic factors, with community advisory board members and staff from the local communities participating in interpretation of results and decisions about recalibration thresholds. Such community-engaged auditing processes ensure that technical performance metrics are evaluated against community values regarding acceptable trade-offs between different types of errors.

## Example of Community Engagement to Adapt and Utilize an AI/ML Screening and Self-Management-Support Chatbot

English- and Spanish-speaking Latino populations in Colorado experience disparities in screening, timely diagnoses, and access to treatment for a range of chronic health concerns (Thorpe et al., n.d.). Increasing access to screening, timely diagnosis and treatment, and engagement in self-management activities are recommended strategies to address these inequities. Text-message-based artificially intelligent (AI) conversational chatbots are emerging as a potential tool to facilitate this access.

**Fig. 3:**
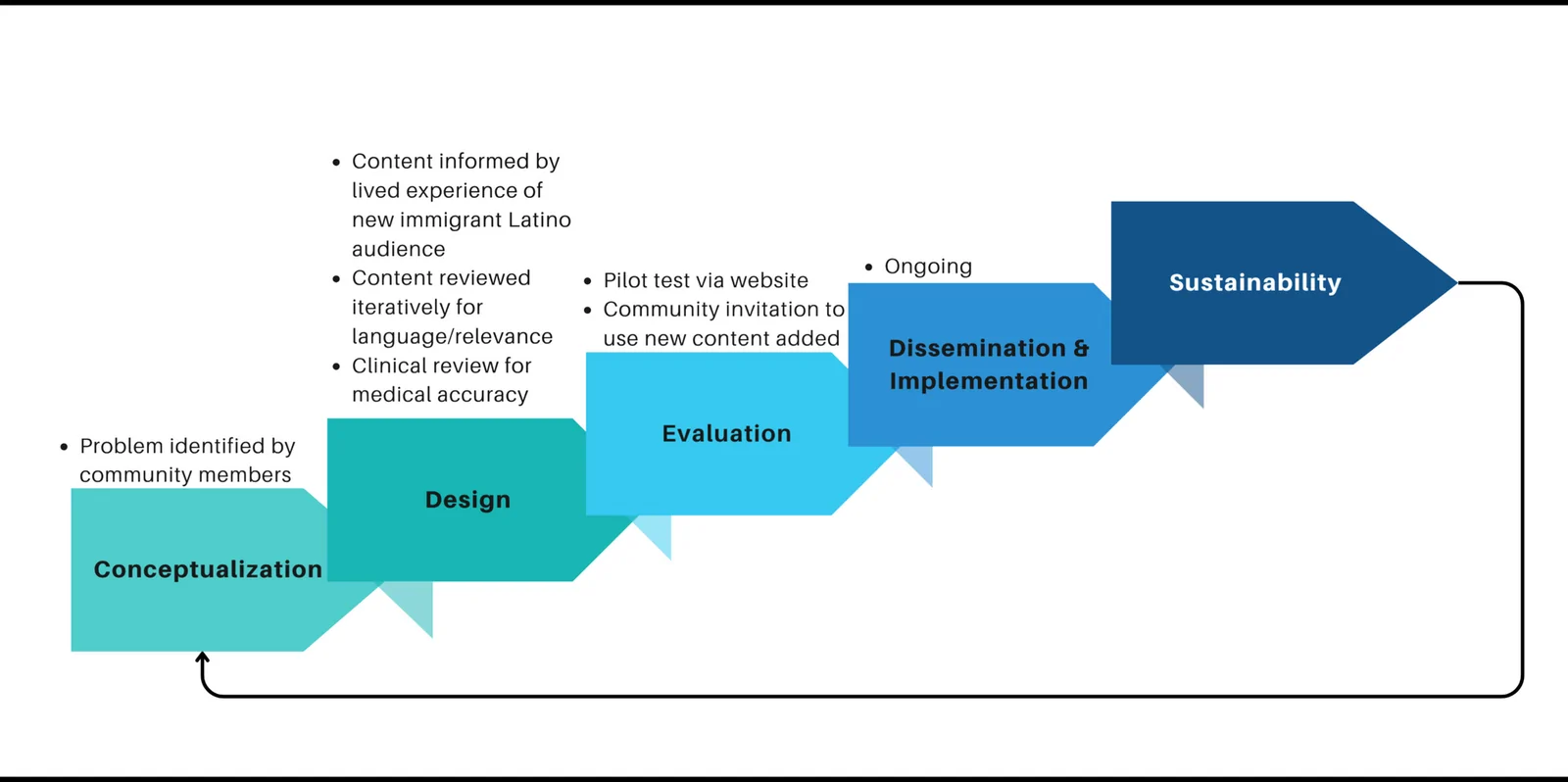
Community engagement in development of the Tepeyac Chatbot

However, there are complexities in working across languages and cultures, and we have little data about their acceptability and feasibility in these communities. Our work aimed to explore how an AI-chatbot could be co-developed with Latino communities. The goal was to adapt existing AI-chatbots designed to facilitate access to screening and self-management for diabetes, hypertension, high cholesterol, and cancer that would resonate for a primarily Spanish-speaking, new immigrant, low-income patient population seeking care through a federally qualified health center, Tepeyac Community Health Center (Tepeyac) (“Tepeyac Community Health Center” (Tepeyac n.d.) (Fig. 3).

**Fig. 4:**
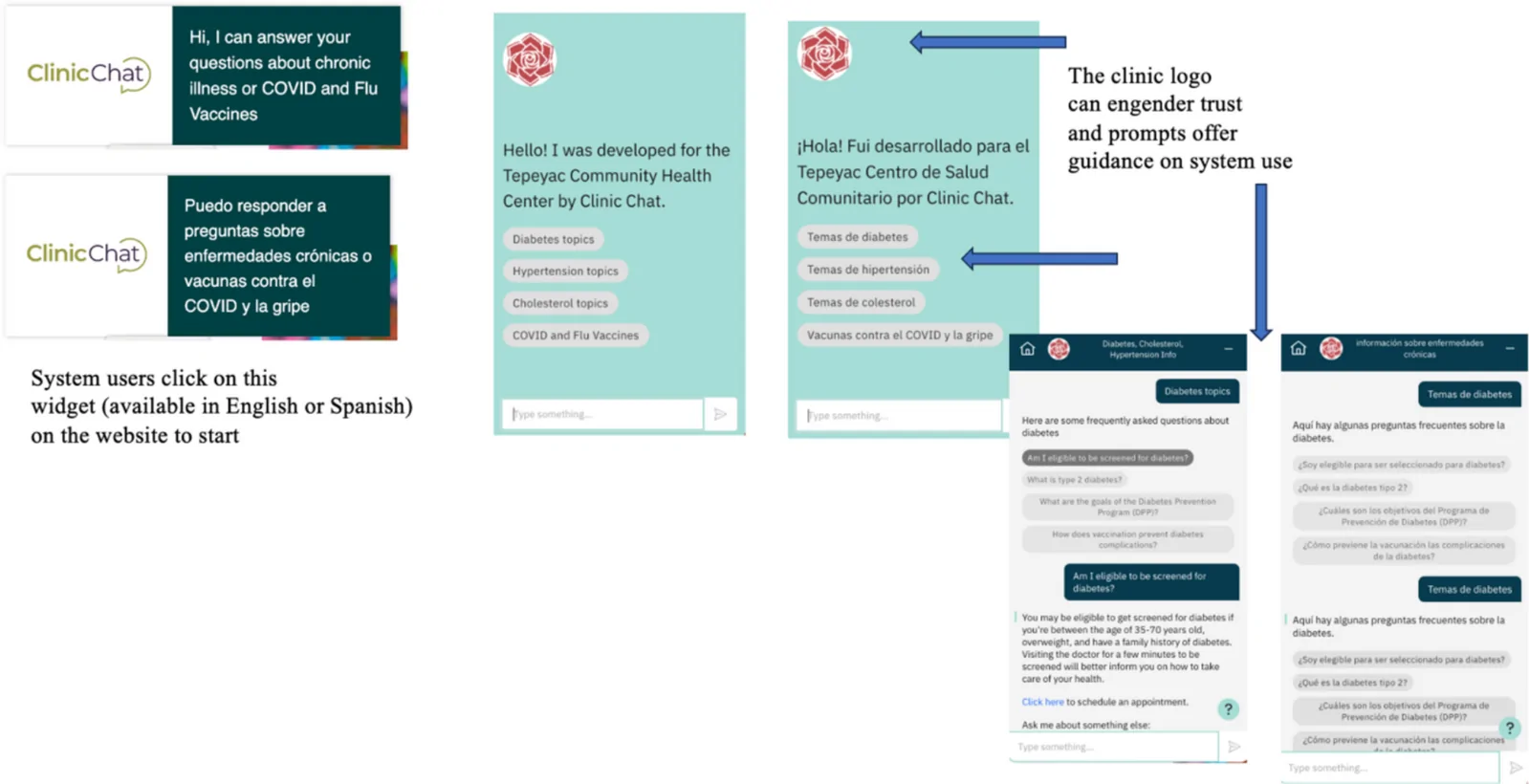
User interface for the AI-chatbot based on community feedback and preferences

We used an iterative design and development process. During the Conceptualization and Design phases, we collaborated with two local programs “Ventanillas de Salud” and Servicios de La Raza, programs that offer in-person blood-pressure and blood-sugar screening and referrals for care to people seeking consular services. We conducted in-depth interviews with *N* = 18 of these programs’ clients and asked them if they were familiar with chatbots; what they thought of them; whether chatbots could be used to access information and support for healthy behaviors; and inquired about strategies to encourage use of chatbots. We learned that any chatbot system would need to be branded with the name of a healthcare organization to demonstrate endorsement and engender trust; to be offered in Spanish; to offer users prompts and shortcuts to help users understand the system and minimize typing; and to include relatable content to encourage engagement and ongoing use. These suggestions were integrated into the Chatbot system. For example, if a client asked about physical activity, the Chatbot would include suggestions of types of movement that resonate more for Latinos based on our qualitative interviews, such as Dancing (salsa, merengue, hip hop!), bird watching, and play tag with the user’s children (see Appendix Table 1 and Fig. 4 for further examples).

The Design Phase involved generating, categorizing, and creating variations for each anticipated “intent” (i.e., the specific topics we believed people wanted to learn or ask about screening, diagnosis, and treatment), designing the system to revert to a fixed choice of responses if no match was found, and using data-augmentation techniques to update the system’s library of questions and variations. Additionally, the provider team at Tepeyac was consulted for medical accuracy and cultural relevance.

The Evaluation Phase the chatbot utilized natural language processing with intent classification algorithms achieving 78% accuracy in matching user queries to appropriate responses. The technical architecture employed approaches similar to those described in Bull et al. (2024, 2025) for health-focused chatbot development, with full technical specifications to be detailed in future publications.

Access to the system was made available on the Tepeyac website on August 15, 2023, and remains functional today. As part of ongoing monitoring, we log all engagements with the chatbot, track each communication, and log incorrect responses. As of January 1, 2024, there were 554 queries to the system from 172 unique users. The average number of messages per user was 3.2, suggesting people ask multiple questions.

The chatbot implementation utilizes the following ethical safeguards: users access this system without any login requires and do so anonymously when accessing the chatbot on a website. The system records user IP addresses, which can be considered protected health information (PHI), although an IP address is not necessarily unique for a user who shares a computer. Users are notified they should call 911 for a medical emergency or 988 for a mental health emergency. Every user who accesses the system are notified upon first accessing the system that the intent of the chatbot is to provide general information only.

Users are informed upon first accessing the system that logs documenting questions and answers are retained only on the clinic servers behind a secure firewall. Decisions as to whether and how long to keep these logs are the purview of clinics, because they represent patient engagement and may inform clinical decision-making regarding patient education in the future.

This work has been classified as exempt from IRB review, given the emphasis on patient education and the minimal risk for privacy or confidentiality issues and thus represents an example of how clinics might use an AI chatbot for quality improvement.

## Example of Community Engagement to Develop an AI/ML Tool for Community Health Workers

As an example of deep engagement during Conceptualization Phase, in the Spring of 2022, Kula no nā Po’e Hawai’i o Papakōlea, Kewalo, Kalāwahine (KULA), a Hawai’i community-based nonprofit, sought to develop an AI/ML tool for Native Hawaiian/Pacific Islander (NHPI) populations Community Health Workers (CHWs). The goal is to draw upon KULA datasets to address health disparities within the Hawaiian homestead region of Papakōlea. Specifically, KULA’s aim is to apply AI/ML to the Native Hawaiian Homestead Health Survey (HHS) dataset to create an AI-powered application for CHWs to improve program coverage and resource recommendations.

The project is rooted in a cultural safety research praxis, HILINA'I, a model of community-based safety for Indigenous and communities of color that builds trust and cultural safety when engaging in research (Ka‘opua et al., 2017). KULA uses this framework to ensure that the community of Papakōlea is engaged on their own terms to advance self-sufficiency and self-determination while preserving the values, traditions, and Native Hawaiian culture. The project is driven by the concept: “No kakou, na kakou.”

During the Conceptualization Phase, a Community Advisory Board was formed to guide activities. The CHWs and KULA staff were introduced to AI/ML through training and a group modeling exercise with feedback gathered throughout the workshop. This information, coupled with a larger process involving the National Association of Pasifika Organizations (NAOPO) that relied on “*talanoa*—talk story” sessions held with 15 local organizations to gather information on communities’ knowledge of and experience and attitudes towards using AI/ML, has yielded important community-driven lessons, including: (1) Prioritize needs as perceived by the community itself; (2) Establish relationships to foster trust, understanding, and address cultural safety concerns; (3) Develop sustainable and practical solutions to health inequities by incorporating the wisdom, knowledge, and experiences of community members, leaders, and advocates; (4) Engage with the community from the outset to facilitate transparent collaboration; (5) Collaborate with the community to create solutions that are truly meaningful to them, rather than imposing preconceived notions; (6) Encourage both the community and AI/ML experts to invest in learning each other’s “language” to facilitate effective communication and understanding; (7) Foster reciprocal relationships and concurrent learning among all research partners; and (8) Humanize the process of ML model building to make it comprehensible and engaging for the community, thereby increasing their willingness to participate. These lessons are foundational to approaches to engage communities in helping to develop AI/ML tools that will most benefit communities with the highest needs. Technical specifications for the planned AI/ML application will be detailed in future publications as the project advances through implementation phases.

## Conclusion

Our framework demonstrates how community engagement can be systematically integrated across all phases of tool development for AI/ML-driven prevention efforts. Our case studies illustrate emerging approaches. Each example demonstrates different approaches: Native-RISE’s focus on community-driven priorities and expansion to health systems, the chatbot’s adaptation across languages and cultural contexts, and KULA’s community-driven development. These diverse pathways suggest that successful implementation, sustainability and scaling requires maintaining core community engagement principles while adapting methods to local contexts, infrastructure, and community capacity.

Community engagement is critical to developing AI/ML tools for use in prevention science. By incorporating diverse perspectives at every stage of model development and implementation, these tools become more closely aligned to real-world needs and challenges of end-users. Authentic community engagement takes time but engenders trust. Ultimately, integrating community insights into AI/ML enabled prevention tools is not just a matter of enhancing functionality but is essential in advancing health equity.

## Appendix

**Table 1:** Examples of changes to chatbot messages to increase cultural relevance and engagement

Topic (English)	Topic (Spanish)	English response	Spanish response	How culturally tailored?
How can I make physical activity more fun?	¿Cómo puedo hacer que la actividad física sea más divertida?	Dancing (salsa, merengue, hip hop!), walking with a friend, and bird watching are all things you can do to stay active. If you have kids in your life, playing tag and going to the playground work, too.	Bailar salsa, merengue, hip hop!; Caminar con un amigo y observar el paisaje a tu alrededor, es todo lo que puedes hacer para mantenerte activo. Si tienes hijos ," el juego de la mancha" o ir con ellos al parque de juegos, tambien es una buena opcion!.	Focus is on types of movement that resonate for Latinos
How do I manage my blood sugar levels?	¿Cómo manejo mis niveles de azúcar en la sangre?	Did you know that both Halle Berry and Tom Hanks both have type II diabetes? This means they have to control what they eat and make sure to stay active as two key ways to manage their blood sugar and keep it at a healthy level. I can help you with ideas on how--just ask!	¿Sabías que tanto Isabel Pantoja, la Jueza Sotomayor como Don Francisco tienen diabetes tipo II? Esto significa que tienen que controlar lo que comen y asegurarse de mantenerse activos como tambien de controlar su azúcar en la sangre y mantenerlo en un nivel saludable. Puedo ayudarte con ideas sobre cómo lograrlo ¡solo pregunta!	Role models identified in the English content are different than the Spanish content; the latter includes people better known to the Latino community
What are some steps I can take to reduce stress?	¿Cuáles son algunos pasos que puedo tomar para reducir el estrés?	You might know people like Maria & Jorge who have promised their kids that they will have a family dinner at their house every week—even if it is just to share sopa. You can make this plan too as a way to reduce stress.	Posiblemente conoces a María , una gran líder de Colorado, que tiene un saludable habito para reducir el estres. Maria suele planear junto con sus hijos ,una cena familiar al menos una vez al mes y con sus amigos mas cercanos , se reunen para charlar durante los fines de semana!. También puedes hacer este plan como una forma de reducir el estrés.	Focus is on types of activities and food that can resonate for diverse communities
How can I access a cancer support group in the Denver area?	¿Cómo puedo acceder a un grupo de apoyo para pacientes con cáncer en Denver?	People living in the Denver area have a couple of choices for support groups. Will you commit to one of these today? The XXX Church located at XXX., Arvada, CO offers a cancer support group. Also, Coffee & Conversations is an online option. Contact XXX at (303) XXX-XXXX.	Las personas que viven en Colorado tienen varias opciones en grupos de apoyo. ¿Te comprometerás con uno de esos grupos hoy? Haga clic aquí para obtener información sobre grupos de apoyo en español.	The support group identified in the English content does not offer options for exclusive Spanish speakers; we tailored information for the Spanish speakers to access support in their preferred language
